# Proposal and multicentric validation of a laparoscopic Roux-en-Y gastric bypass surgery ontology

**DOI:** 10.1007/s00464-022-09745-2

**Published:** 2022-10-26

**Authors:** Joël L. Lavanchy, Cristians Gonzalez, Hasan Kassem, Philipp C. Nett, Didier Mutter, Nicolas Padoy

**Affiliations:** 1 IHU Strasbourg, 1 Place de l’Hôpital, 67000 Strasbourg, France; 2 Department of Visceral Surgery and Medicine, Inselspital, Bern University Hospital, University of Bern, Bern, Switzerland; 3 University Hospital of Strasbourg, Strasbourg, France; 4 ICube, CNRS, University of Strasbourg, Strasbourg, France

**Keywords:** Laparoscopic Roux-en-Y gastric bypass, Ontology, Inter-rater reliability, Intra-rater reliability, Surgical data science

## Abstract

**Background:**

Phase and step annotation in surgical videos is a prerequisite for surgical scene understanding and for downstream tasks like intraoperative feedback or assistance. However, most ontologies are applied on small monocentric datasets and lack external validation. To overcome these limitations an ontology for phases and steps of laparoscopic Roux-en-Y gastric bypass (LRYGB) is proposed and validated on a multicentric dataset in terms of inter- and intra-rater reliability (inter-/intra-RR).

**Methods:**

The proposed LRYGB ontology consists of 12 phase and 46 step definitions that are hierarchically structured. Two board certified surgeons (raters) with > 10 years of clinical experience applied the proposed ontology on two datasets: (1) StraBypass40 consists of 40 LRYGB videos from Nouvel Hôpital Civil, Strasbourg, France and (2) BernBypass70 consists of 70 LRYGB videos from Inselspital, Bern University Hospital, Bern, Switzerland.

To assess inter-RR the two raters’ annotations of ten randomly chosen videos from StraBypass40 and BernBypass70 each, were compared. To assess intra-RR ten randomly chosen videos were annotated twice by the same rater and annotations were compared.

Inter-RR was calculated using Cohen’s kappa. Additionally, for inter- and intra-RR accuracy, precision, recall, F1-score, and application dependent metrics were applied.

**Results:**

The mean ± SD video duration was 108 ± 33 min and 75 ± 21 min in StraBypass40 and BernBypass70, respectively. The proposed ontology shows an inter-RR of 96.8 ± 2.7% for phases and 85.4 ± 6.0% for steps on StraBypass40 and 94.9 ± 5.8% for phases and 76.1 ± 13.9% for steps on BernBypass70. The overall Cohen’s kappa of inter-RR was 95.9 ± 4.3% for phases and 80.8 ± 10.0% for steps. Intra-RR showed an accuracy of 98.4 ± 1.1% for phases and 88.1 ± 8.1% for steps.

**Conclusion:**

The proposed ontology shows an excellent inter- and intra-RR and should therefore be implemented routinely in phase and step annotation of LRYGB.

**Supplementary Information:**

The online version contains supplementary material available at 10.1007/s00464-022-09745-2.

The aim of Surgical Data Science (SDS) is to analyze data sources acquired during surgical treatment to improve patient safety and clinical outcomes [1]. Opposed to traditional clinical research centering on preoperative patient characteristics and postoperative outcomes, SDS focuses on the whole data stream of surgical treatment. To unravel the “black box” of the operation room (OR) and the impact of the understudied intraoperative phase on patient outcomes, SDS particularly analyzes data streams captured in the OR during surgery.

Since the introduction of video technology in minimally invasive surgery, video recordings of surgical interventions are easily recorded and therefore readily available. As analysing surgical videos is time consuming, costly, and often lacks objectivity, the full potential of video analysis was often not tapped in the past [2]. In the last decades however, the evolution of computer vision (CV), which is the analysis of visual information by computer algorithms, boosted the potential of surgical video analysis.

One of the most analysed surgeries in SDS is laparoscopic cholecystectomy (LCHE). Classical CV tasks in the analysis of surgical videos are phase recognition and tool presence detection. They were developed for LCHE [3]. Moreover, safety feedback [4, 5] and surgical skill assessment algorithms [6] were trained on LCHE videos. However, the disadvantage of LCHE as a model intervention for SDS is that there are hardly any intraoperative events or postoperative complications to study given the limited size of datasets. Therefore, recent SDS research focuses on longer and more complex procedures like colorectal [7, 8] or bariatric surgery [9, 10].

Laparoscopic Roux-en-Y gastric bypass (LRYGB) is one of the most performed bariatric surgeries worldwide [11, 12]. The reported numbers of postoperative complications range between 4 and 13% [13–15]. Its technical standardization, the moderate duration, and frequent postoperative outcome events, make it an excellent candidate for CV-assisted video analysis.

Surgical interventions can be hierarchically decomposed into phases (e.g., access, mobilization, resection, reconstruction, disassembling), that consist of more fine-grained steps (e.g., cavity exploration, trocar placement, retractor placement, etc.) [16]. In contrast to the technical standardization an ontology defines how to describe a surgical intervention in a structured and generic way [16, 17]. The word ontology is derived from the ancient Greek words ὄν (being, that which is) and λόγος (reason, rational, principle, logical reasoning) and refers to the ‘study of being’.

However, most datasets in SDS are small, monocentric, and lack external validation. To overcome these limitations, larger and multicentric datasets are warranted. Furthermore, variability of ontologies and its application on datasets limits generalization across centers [18]. To ensure data quality and reliable algorithms procedure-specific ontologies need to be defined and validated multicentrically.

This work is the first to propose a LRYGB ontology for phases and steps and to validate it on a multicentric dataset in terms of inter- and intra-rater reliability. The application of this ontology enables workflow analysis across surgeons and centers. Furthermore, it facilitates downstream applications of SDS, as the training of phase and step recognition algorithms using artificial intelligence for automated surgical video analysis.

## Methods

### Ontology

**Fig. 1:**
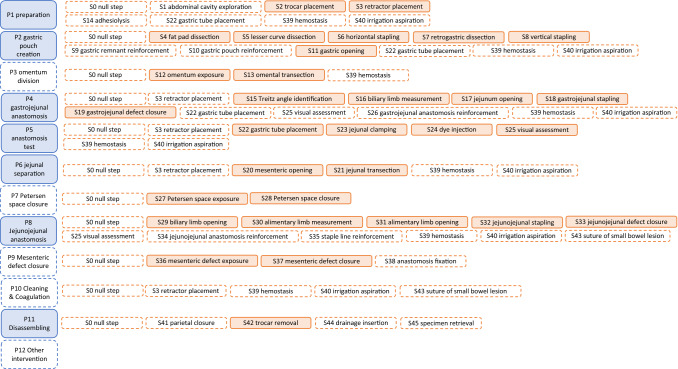
Hierarchical structure of phases and steps in the proposed laparoscopic Roux-en-Y gastric bypass ontology. Facultative phases and steps have a dashed border

The proposed LRYGB ontology was developed by the surgical staff of the Department of Digestive and Endocrine Surgery at Nouvel Hôpital Civil (University Hospital of Strasbourg), France [10]. Based on pre-recorded anonymized surgical videos, the procedure was hierarchically broken down into phases and steps. Phases were defined as all first level temporal components that must be executed sequentially to allow the achievement of the surgical objectives. While the steps were defined as the set of actions that must be accomplished during the phases to yield the task of choice. The hierarchical structure of the ontology is displayed in Fig. 1. Subsequently, a temporal annotation framework to define the start and end time of each phase and each step was agreed upon by consensus. This ontology was presented, discussed, and validated by a panel of international faculty that attended the Laparoscopic and Endoluminal Bariatric and Metabolic Surgery Course held at IRCAD France from November 28 to December 01, 2018. It was adapted for multicentric use and contains 12 phase and 46 step definitions as outlined in the Supplementary Material (Tables S1, S2).

### Datasets

Two board certified visceral surgeons (referred to as raters) with over 10 years of clinical expertise applied the proposed ontology to two datasets. (1) The StraBypass40 dataset consists of 40 LRYGB videos recorded at Nouvel Hôpital Civil, University Hospital of Strasbourg, France [10]. (2) The BernBypass70 dataset consists of 70 LRYGB videos recorded at Inselspital, Bern University Hospital, Bern, Switzerland.

### Intervention

Inter-rater reliability (inter-RR) defines the extent of agreement among observers, whereas intra-rater reliability (intra-RR) defines the consistency of observations of a given observer over time. To assess the inter-RR of the proposed LRYGB ontology ten randomly chosen videos of the StraBypass40 and BernBypass70 datasets were annotated according to the step and phase definitions as provided in the Supplementary Material (Tables S1, S2) by both raters using the in-house video annotation tool MOSaiC. The annotations of both raters were compared. To assess the intra-RR of the proposed LRYGB ontology ten randomly chosen videos were annotated a second time by the same rater after a wash out phase of 1 month. The two sets of annotations were compared.

### Evaluation

Inter- and intra-RR was calculated using accuracy, precision, recall and F1-scores. Accuracy is the proportion of correct predictions among the total number of observations. Precision is the proportion of true positives among all (true and false) positives and referred to as the positive predictive value. Recall is the proportion of true positives among all relevant observations (true positives and false negatives) and referred to as sensitivity. F1-score is the harmonic mean of precision and recall and is a measure of accuracy.

Furthermore, average transitional delay, noise level and a coefficient of transitional moments were calculated to apply application dependent metrics as proposed in [19]. Every transition from phase to phase or from step to step is considered a transitional moment. Average transitional delay is the average delay between the annotated and the real transitional moment. It can be positive or negative. Noise level is the proportion of annotated phases or steps not being part of a real transitional moment among all annotated phases or steps. The coefficient of transitional moments is the ratio of annotated to real transitional moments. The transitional delay threshold was set to 5 s. Cohen’s kappa has been used to calculate inter-rater reliability to account for agreement of raters by chance [20].

The comparison of two sets of annotations is not symmetric. In the validation of computer algorithms, the human annotation always serves as ground truth. Given that this study compares two set of human annotations, each set was treated once as ground truth and metrics were averaged across both comparisons. All metrics were applied for every video separately on phases and steps on a millisecond level and averaged across datasets.

## Results

For StraBypass40 the mean ± SD video duration was 108 ± 33 min An average LRYGB video consisted of 10 phases and 33 steps.

For BernBypass70 the mean ± SD video duration was 75 ± 21 min An average LRYGB video consisted of 8 phases and 27 steps.

**Fig. 2:**
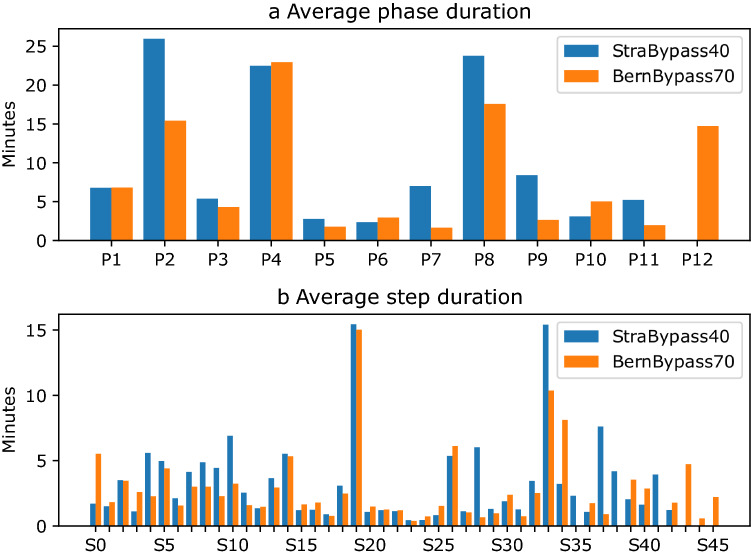
Average duration of **a** phases and **b** steps. The labels correspond to the respective phase / step as outlined in Fig. 1

Average phase and step durations in the StraBypass40 and BernBypass70 datasets are displayed in Fig. 2.

**Fig. 3:**
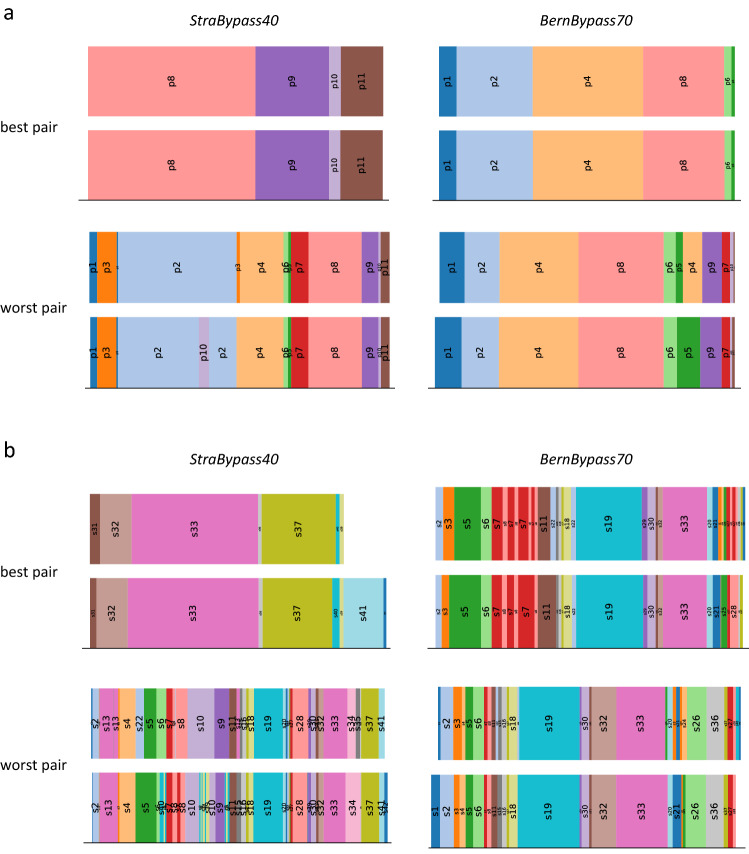
Visual comparison of annotations. **a** Phase annotation, **b** Step annotation. In the top row comparison of the best matching annotation pairs, in the bottom row comparison of the worst matching annotation pairs of the StraBypass40 and BernBypass70 datasets. The width of each phase / step corresponds to its relative duration and the labels correspond to the respective phase / step as outlined in Fig. 1

Quantitative inter-RR results for StraBypass40, BernBypass70 and overall inter-RR results are shown in Table 1. Intra-RR results are shown in Table 2. Qualitative results in form of a visual comparison of the best and worst matching phase and step annotation pairs for StraBypass40 and BernBypass70 are shown in Fig. 3.

**Table 1:** Validation of the laparoscopic Roux-en-Y gastric bypass ontology: Inter-rater reliability results

		Cohen’s kappa	Accuracy	Precision	Recall	F1-score	ATD (seconds)	C_TM_	NL	AD-accuracy	AD-precision	AD-recall	AD-F1-score
Bypass40	Phases	96.8 ± 2.7	97.4 ± 2.1	92.9 ± 6.6	92.9 ± 6.6	92.6 ± 6.9	8.2 ± 4.8	100.1 ± 0.2	1.5 ± 2.3	97.8 ± 2.2	93.8 ± 6.8	93.8 ± 6.8	93.5 ± 7.1
	Steps	85.4 ± 6.0	86.6 ± 6.0	74.2 ± .10.4	74.2 ± 10.4	72.0 ± 10.0	13.4 ± 9.0	100.9 ± 1.0	9.2 ± 6.4	87.9 ± 6.2	77.1 ± 10.8	77.3 ± 11.1	75.2 ± 10.7
Bypass70	Phases	94.9 ± 5.8	96.0 ± 4.6	86.6 ± 12.0	86.6 ± 12.0	85.8 ± 12.0	10.0 ± 3.3	100.8 ± 1.9	2.5 ± 4.9	96.5 ± 4.7	89.0 ± 10.8	88.4 ± 12.4	87.8 ± 12.3
	Steps	76.1 ± 13.9	78.4 ± 12.6	58.8 ± 20.5	58.8 ± 20.5	57.2 ± 19.7	10.7 ± 6.2	101.4 ± 0.6	18.0 ± 14.3	79.6 ± .13.2	61.3 ± 21.4	61.4 ± 21.7	59.8 ± 20.9
Overall	Phases	95.9 ± 4.3	96.7 ± 3.7	89.7 ± 10.1	89.7 ± 10.1	89.2 ± 10.4	9.1 ± 4.2	99.1 ± 5.7	2.0 ± 3.9	97.1 ± 3.7	91.5 ± 9.4	91.0 ± 10.2	90.7 ± 10.5
	Steps	80.8 ± 10.0	82.5 ± 10.7	66.5 ± 18.1	66.5 ± 18.1	64.6 ± 17.3	12.4 ± 7.6	100.4 ± 10.7	13.7 ± 11.9	83.7 ± 11.0	69.2 ± 18.9	69.4 ± 18.9	67.6 ± 18.3

All reported metrics are mean ± SD, in percent if not stated otherwise

*ATD* average transitional delay, *C*_TM_ coefficient of transitional moments, *NL* noise level, *AD* application dependent

**Table 2:** Validation of the laparoscopic Roux-en-Y gastric bypass ontology: Intra-rater reliability results

	Accuracy	Precision	Recall	F1-Score	ATD (seconds)	C_TM_	NL	AD-accuracy	AD-precision	AD-recall	AD-F1-score
Phases	98.4 ± 1.1	95.3 ± 4.4	95.3 ± 4.4	95.2 ± 4.4	7.8 ± 5.2	100.7 ± 1.9	0.3 ± 0.6	98.8 ± 1.0	96.5 ± 4.4	96.6 ± 4.4	96.5 ± 4.4
Steps	88.1 ± 8.1	77.8 ± 16.4	77.8 ± 16.4	76.5 ± 16.2	6.7 ± 3.2	100.3 ± 0.4	8.2 ± 8.6	89.5 ± 8.4	81.1 ± 16.8	81.0 ± 17.5	79.7 ± 17.3

All reported metrics are mean ± SD, in percent if not stated otherwise

*ATD* average transitional delay, *C*_TM_ coefficient of transitional moments, *NL* noise level, *AD* application dependent

Across datasets inter-RR and intra-RR metrics show better results for phase compared to step recognition. Furthermore, inter-RR metrics on StraBypass40 show better results than on BernBypass70. The application dependent metrics show a 0.4–1.2% boost compared to the classical metrics.

## Discussion

The excellent inter-RR of 95.9% for phases and 80.8% for steps of the proposed ontology demonstrate its easy application and reliable use for the annotation of LRYGB phases and steps by multiple raters in multiple institutions. Moreover, the excellent intra-RR of 98.4% for phases and 88.1% for steps shows that annotations of the same rater are consistent over time.

Given these tremendous results, we advocate for the routine use of the proposed ontology in LRYGB. This will standardize video analysis of LRYGB surgery and will allow comparison of surgical workflows across surgeons and centers. The routine use of this ontology facilitates standardized video review for educational purposes, performance assessment, and quality improvement programs. Furthermore, it enables downstream applications as the training of artificial intelligence algorithms to automatically recognize phases and steps, to give intraoperative feedback or assistance.

For laparoscopic cholecystectomy, one of the most analyzed surgeries in SDS, a systematic review identified 8 different phase definitions in the literature [18]. Multiple phase definitions are a hindrance to comparison of results across datasets and institutions. Therefore, with the definition and multicentric validation of an LRYGB phase and step ontology we aim to prevent the use of multiple competing ontologies. To implement the use of the proposed ontology on a global scale, awareness for surgical video recording in general, and in particular, larger consensus among bariatric surgeons using the Delphi method must be created.

Inter-RR and intra-RR metrics are higher for phase compared to step annotation. Comprising 12 phases and 46 steps the proposed ontology is less granular on the phase than on the step level. This leads to lower variability in phase compared to step annotations. Therefore, the ontology performs better in terms of inter- and intra-RR on a phase than on a step level.

As the two datasets are from different institutions and therefore represent different surgical techniques, the aim of this study is not to compare them. However, to understand the performance difference of the proposed ontology on StraBypass40 and BernBypass70 it is crucial to elaborate, how they differ. When comparing StraBypass40 with BernBypass70*,* there is a considerable difference in average video duration (108 vs. 75 min). This is also reflected by the greater average number of phases and steps in StraBypass40 compared to BernBypass70 (10 vs. 8 phases, 33 vs. 27 steps). In StraBypass40 the creation of the gastric pouch (phase 2, 26 vs. 15 min) and the creation of the jejunojejunal anastomosis (phase 8, 24 vs. 18 min) takes considerably longer when compared to BernBypass70. The main differences in surgical technique between datasets are the routine division of the omentum (phase 3, 95 vs. 36%), Petersen space (phase 7, 98 vs. 16%) and mesenteric defect closure (phase 9, 100 vs 21%) in StraBypass40 compared to BernBypass70*.*

Inter-RR metrics on StraBypass40 show better results when compared to BernBypass70. This is likely an effect of the difference in average video duration between datasets. Given the same number of phase and step transitions, the longer a video is, the less the metrics are influenced by a single transitional delay.

Using application dependent metrics, a 0.4–1.4% boost in accuracy, precision, recall and F1-score can be observed. This is due to the relaxation of transitional moments by extension of the acceptable transitional delay. Setting the transitional delay threshold allows to tailor the metrics application dependent to the desired use case. To estimate the remaining time of an intervention based on a phase and step recognition algorithm a 5 s delay is reasonable. However, for real-time intraoperative decision support 5 s delay are too long and will limit the acceptance of the application. Considering the first use case, in this study the transitional delay threshold was set at 5 s to calculate application dependent metrics as proposed in [19].

### Limitations

Despite being multicentric this study includes only two raters and two institutions. As surgical video annotation is time consuming and needs domain expertise, it is expensive. Therefore, annotation resources must be attributed carefully.

### Conclusion

The proposed ontology shows an excellent inter- and intra-RR and should therefore be implemented routinely in phase and step annotation of LRYGB videos. This will facilitate education, performance assessment, and quality improvement programs. Moreover, the application of the proposed ontology will enable the development of downstream tasks as automated phase and step recognition, intraoperative feedback, or assistance by use of artificial intelligence.

## Supplementary Information

Below is the link to the electronic supplementary material.

Supplementary file1 (PDF 130 kb)
