# Supplementary material for: Proposal and multicentric validation of a laparoscopic Roux-en-Y gastric bypass surgery ontology
Source: Surg Endosc. 2022 Oct 26;37(3):2070–7. doi: 10.1007/s00464-022-09745-2 (PMC10017621; doi:10.1007/s00464-022-09745-2)
Supplement: Supplementary file 1 — Supplementary file1 (PDF 130 kb) [file 464_2022_9745_MOESM1_ESM.pdf]

Supplementary material

Proposal and Multicentric Validation of a Laparoscopic Roux-en-Y Gastric Bypass Surgery Ontology

Joël L. Lavanchy MD[1,2], Cristians Gonzalez MD[1,3], Hasan Kassem M.Eng[4], Philipp C. Nett MD[2], Didier Mutter MD PhD[1,3], Nicolas Padoy PhD[1,4]

1 IHU Strasbourg, France

2 Department of Visceral Surgery and Medicine, Inselspital Bern University Hospital, University of Bern, Switzerland

3 University Hospital of Strasbourg, France

4 ICube, University of Strasbourg, CNRS, France

Correspondence to:

Joël L. Lavanchy MD, IHU Strasbourg

1 Place de l’hôpital, 67000 Strasbourg, France

joel.lavanchy@ihu-strasbourg.eu

Table S1: Definition of the phases of laparoscopic Roux-en-Y gastric bypass

ID	Phase name	Description	Start of the phase	End of the phase
P1	Preparation	Access to the abdominal cavity, installation of the ports and exposure of the operating field	Introduction of the camera into the abdomen	Placement of the liver retractor
P2	Gastric pouch creation	The proximal part of the stomach is separated from the rest to create a gastric pouch	Dissection of the fat pad around the esophagogastric junction	Opening of the gastric pouch
P3	Omentum division	Vertical transection of the omentum majus to facilitate the ascent of the small bowel to the gastric pouch	Grasping of the omentum	Omentum fully transected at the level of the transverse colon
P4	Gastrojejunal anastomosis	Anastomosis of the small bowel with the gastric pouch	Exposure and visualization of the Treitz angle	Completion of gastrojejunal closure
P5	Anastomosis test	Verification that the gastrojejunostomy does not leak	Clamping of the jejunum distal to the gastrojejunostomy	Visual assessment of the anastomosis
P6	Jejunal separation	Separation of the proximal alimentary limb and the biliary limb by transection of the jejunum	Grasping the mesentery to open it	Removal of the stapler after transection of the jejunum
P7	Petersen space closure	Closure of the Petersen space between the alimentary limb and the transverse mesocolon	Exposure of the Petersen space	Closure of the Petersen space
P8	Jejunojejunal anastomosis	Anastomosis of the distal alimentary limb with the biliary limb	Opening of the biliary limb	Completion of the jejunojejunal closure

P9	Mesenteric defect closure	Closure of the mesenteric defect at the jejunojejunostomy	Exposure of the mesenteric defect	Closure of the mesenteric defect
P10	Cleaning & coagulation	Irrigation and aspiration of liquid / blood in the abdominal cavity, hemostasis	Introduction of the aspiration/irrigation device and / or the hemostasis instruments	Removal of the aspiration/irrigation device and / or the hemostasis instruments
P11	Disassembling	Removal of the surgical instruments, retractor, ports and camera	Removal of the surgical instruments	Removal of the camera
P12	Other intervention	If an additional intervention is performed (e.g. liver biopsy, cholecystectomy)	Beginning of the additional intervention	End of the additional intervention

Table S2: Definition of the steps for laparoscopic Roux-en-Y gastric bypass

ID	Step name	Description	Start of the step	End of the step
S0	Null step	The camera is static and no actions are performed	Termination of a surgical action	Beginning of a surgical action
S1	Abdominal cavity exploration	The abdominal cavity is explored to detect alterations that could modify or prevent the planned surgery	Introduction of the camera to the abdominal cavity	Completion of the abdominal cavity exploration
S2	Trocar placement	Accessory trocars are introduced into the abdominal cavity	Introduction of the first trocar	Introduction of the last trocar
S3	Retractor placement	Introduction and placement of a liver retractor to expose the esophagogastric junction	Introduction of the liver retractor	Exposure of the esophagogastric junction
S4	Fat pad dissection	Dissection of the fatty tissue surrounding the esophagogastric junction to expose the angle of his and remove adhesions to the spleen	Grasping the fat pad at the esophagogastric junction	Exposure and mobilization of the gastric fundus
S5	Lesser curvature dissection	Opening of a retrogastric window at the lesser curvature of the stomach to facilitate the passage of the stapler	Grasping the lesser curvature	Free and easy passage of the grasper to the retrograstric space

S6	Horizontal stapling	Horizontal transection of the stomach with the stapler starting from the lesser curvature to create the horizontal part of the pouch	Introduction of the stapler into the abdominal cavity	Removal of the stapler after complete transection of the horizontal part of the pouch
S7	Retrogastric dissection	Dissection of the tissue dorsal to the stomach for better exposure	Introduction of the grasper exposing and / or the energy device dissecting the retrogastric dissection plane	Removal of the energy device after exposure of the retrogastric space
S8	Vertical stapling	Vertical transection of the stomach with the stapler to create the vertical portion of the pouch	Introduction of the stapler into the abdominal cavity	Removal of the stapler after complete transection of the vertical portion of the pouch
S9	Gastric remnant reinforcement	Reinforcement of the gastric remnant staple line with a suture	Introduction of the suture	Removal of the needle
S10	Gastric pouch reinforcement	Reinforcement of the gastric pouch staple line with a suture	Introduction of the suture	Removal of the needle
S11	Gastric opening	Creation of an orifice into the gastric pouch where the gastrojejunostomy will be created	Introduction of the energy device	Introduction of an instrument into the gastric pouch
S12	Exposure of the omentum	Grasping and lifting of the omentum to expose it	Grasping of the omentum	Exposure of the omentum

S13	Omental transection	Transection of the omentum to divide it into two parts	Introduction of the energy device	Removal of the energy device
S14	Adhesiolysis	Transection of connective tissue or adhesions	Introduction of the cutting instrument	Removal of the cutting instrument
S15	Treitz angle identification	Visualization of the Treitz angle to identify the proximal jejunum	Grasping of transverse mesocolon and / or omentum	Grasping of the jejunum at the Treitz angle
S16	Biliary limb measurement	Measurement of the small bowel length from Treitz angle to the future site of the gastrojejunostomy to determine the length of the biliary limb	Grasping of the jejunum at the Treitz angle	Grasping the jejunum at the future site of the gastrojejunostomy
S17	Jejunum opening	Opening of the distal jejunum where the gastrojejunostomy will be created	Introduction of the energy device	Removal of the instrument dilating the orifice
S18	Gastrojejunal stapling	Creation of the gastrojejunostomy using a stapler	Introduction of the stapler	Removal of the stapler
S19	Gastrojejunal defect closure	Closure of the orifice left by the stapler creating the gastrojejunostomy	Introduction of the suture	Removal of the suture
S20	Mesenteric opening	Opening of the mesentery to facilitate the introduction of the stapler	Introduction of the grasper	Dilatation of the hole with an instrument

S21	Jejunal transection	Transection of the jejunum proximal to the gastrojejunostomy	Introduction of the stapler	Removal of the stapler
S22	Gastric tube placement	Movement of the gastric tube (e.g. to calibrate the size of the gastric pouch or the gastrojejunostomy)	Start of gastric tube movement	End of gastric tube movement
S23	Jejunal clamping	Clamping of the jejunum distal to the gastrojejunostomy	Introduction of the grasper	Clamping of the jejunum
S24	Dye injection	Injection of dye (methylene blue) to detect any leakage of the gastrojejunostomy	Clamping of the jejunum	Bulging of the gastrojejunostomy
S25	Visual assessment	Visual inspection of the anastomosis for any leakages	The camera focuses on the anastomosis	The camera moves away from the anastomosis
S26	Gastrojejunal anastomosis reinforcement	Reinforcement of the gastrojejunostomy with an additional suture	Introduction of the suture	Removal of the needle
S27	Petersen space exposure	Exposure of the Petersen space (between the alimentary limb and the transverse colon)	Grasping of the alimentary limb and / or the transverse mesocolon	Full exposure of the Petersen space

S28	Petersen space closure	Closing of the Petersen space with suture or staples	Introduction of the suture or stapler	Removal of the needle or stapler
S29	Biliary limb opening	Opening of the biliary limb where the jejunojejunostomy will be created	Introduction of the energy device	Removal of the instrument dilating the orifice
S30	Alimentary limb measurement	Measurement of the small bowel length from the gastrojejunostomy to the future site of the jejunojejunostomy to determine the length of the alimentary limb	Grasping of the alimentary limb distal to the gastrojejunostomy	Grasping the jejunum at the site of the future jejunojejunostomy
S31	Alimentary limb opening	Opening of the alimentary limb where the jejunojejunostomy will be created	Introduction of the energy device	Removal of the instrument dilating the orifice
S32	Jejunojejunal stapling	Creation of the jejunojejunostomy using a stapler	Introduction of the stapler	Removal of the stapler
S33	Jejunojejunal defect closure	Closure of the orifice left by the stapler creating the jejunojejunostomy	Introduction of the suture	Removal of the suture
S34	Jejunojejunal anastomosis reinforcement	Reinforcement of the jejunojejunostomy with an additional suture	Introduction of the suture	Removal of the needle

S35	Staple line reinforcement	Staple line reinforcement of the blind limb of the jejunojejunostomy with an additional suture	Introduction of the suture	Removal of the needle
S36	Mesenteric defect exposure	Exposure of the mesenteric defect created by the jejunojejunostomy	Grasping of the jejunojejunostomy	Full exposure of the mesenteric defect
S37	Mesenteric defect closure	Closing of the mesenteric defect with suture or staples	Introduction of the suture or stapler	Removal of the needle or stapler
S38	Anastomosis fixation	One or more stiches to fix the position of an anastomosis	Introduction of the suture	Removal of the needle
S39	Hemostasis	Any intervention to stop bleeding	Introduction of the energy device, clip applier, suture, or gauze	Removal of the energy device, clip applier, needle, or gauze
S40	Irrigation / aspiration	Irrigation and aspiration of any liquid or blood clots to be removed from the abdominal cavity	Introduction of the irrigation/aspiration device	Removal of the irrigation/aspiration device
S41	Parietal closure	Closure of the abdominal wall at the trocar sites	Introduction of the Reverdin needle	Removal of the Reverdin needle leaving a suture in place
S42	Trocar removal	Removal of the trocars and the liver retractor	The liver retractor is removed	The camera exits the abdominal cavity

S43	Suture of small bowel lesion	Rectification of a small bowel lesion using a suture	Introduction of the suture	Removal of the needle
S44	Drainage insertion	Insertion of a drainage into the abdominal cavity to drain fluids	Introduction of the drainage	Completion of drainage placement
S45	Specimen retrieval	Removal of any spare tissue (e.g. omentum, small bowel, or stomach)	Grasping the spare tissue	Removal of the tissue or the retrieval bag
